# Use of Non-Invasive Parameters of Non-Alcoholic Steatohepatitis and Liver Fibrosis in Daily Practice - An Exploratory Case-Control Study

**DOI:** 10.1371/journal.pone.0111551

**Published:** 2014-10-28

**Authors:** Karel Dvorak, Jan Stritesky, Jaromir Petrtyl, Libor Vitek, Renata Sroubkova, Martin Lenicek, Vaclav Smid, Martin Haluzik, Radan Bruha

**Affiliations:** 1 Charles University in Prague, 1st Faculty of Medicine, 4th Department of Internal Medicine, Prague, Czech Republic; 2 Charles University in Prague, 1st Faculty of Medicine, Department of Pathology, Prague, Czech Republic; 3 Charles University in Prague, 1st Faculty of Medicine, Institute of Medical Biochemistry and Laboratory Diagnostics, Prague, Czech Republic; 4 Charles University in Prague, 1st Faculty of Medicine, 3rd Department of Internal Medicine, Prague, Czech Republic; RWTH Aachen, Germany

## Abstract

**Background:**

Non-alcoholic fatty liver disease (NAFLD) is the hepatic manifestation of a metabolic syndrome. To date, liver biopsy has been the gold standard used to differentiate between simple steatosis and steatohepatitis/fibrosis. Our aim was to compare the relevance of serum non-invasive parameters and scoring systems in the staging of liver fibrosis and non-alcoholic steatohepatitis (NASH) in patients with NAFLD.

**Methods and Findings:**

A total of 112 consecutive patients diagnosed with NAFLD were included. A liver biopsy was performed on 56 patients. The Kleiner score was used for the staging and grading of the histology. Non-invasive parameters for fibrosis (hyaluronic acid; AST/ALT; fibrosis scoring indexes OELF, ELF, BARD score, APRI, NAFLD fibrosis score); and inflammation (M30 and M65 cytokeratin-18 fragments) were measured and calculated. The same analyses were performed in 56 patients diagnosed with NAFLD, who were not indicated for liver biopsy. Based on the liver histology, NASH was diagnosed in 38 patients; simple steatosis in 18 patients. A cut-off value of 750 U/L of serum M65 discriminated patients with and without NASH with a 80% sensitivity and 82% specificity (95% CI:57–95). Fibrosis stage F0–F2 was present in 39 patients; F3–F4 in 17 patients. Serum concentrations of hyaluronic acid were higher in patients with advanced fibrosis (p<0.01); a cut-off value of 25 µg/l discriminated patients with F3–F4 with a 90% sensitivity and 84% specificity from those with F0–F2 (95% CI:59–99). When applying the non-invasive criteria to those patients without a liver biopsy, NASH could only be diagnosed in 16%; however, advanced fibrosis could be diagnosed in 35% of them.

**Conclusions:**

In patients with NAFLD, non-invasive serum parameters with a high accuracy can differentiate those patients with NASH and/or advanced fibrosis from those with simple steatosis. A substantial portion of those patients not indicated for liver biopsy might have undiagnosed advanced fibrosis.

## Introduction

Non-alcoholic fatty liver disease (NAFLD) represents the hepatic manifestation of a metabolic syndrome, a disease affecting a substantial portion of modern populations [1]. NAFLD comprises a spectrum of conditions from simple steatosis, through non-alcoholic steatohepatitis (NASH), ending with liver cirrhosis. With the epidemic increase in the incidence of obesity, NAFLD has become a serious issue for the foreseeable future.

Whereas simple liver steatosis is regarded as a benign condition, NASH can be a progressive liver disease leading to fibrosis and ultimately to cirrhosis. A liver biopsy has long been the only method for NASH diagnosis and for the staging of liver fibrosis. However, there are several drawbacks associated with this procedure. A liver biopsy is an invasive diagnostic method which is distressing to patients. Although it is generally safe, there is some unavoidable risk of major complications (1−3% of patients) or even fatal ones (0.01% of patients) [2]. Other potential problems include sampling heterogenity [3], [4] and the subjectivity and intra/interobserver variability.

Several non-invasive tools and methods have been developed to predict NASH, or to quantify liver fibrosis without having to resort to a liver biopsy. They are either based on an assessment of single substances, or on the calculation of specific scores. Only a minority of these have been externally validated on large numbers of patients. From the large spectrum of biomarkers tested, cytokeratin-18 fragments have shown the most consistent results for differentiating NASH from steatosis [5].

In addition to the necessity to differentiate NASH from simple steatosis, another necessary goal is the non-invasive assessment of liver fibrosis. For the individual patient, the stage of fibrosis is probably the most important prognostic factor. Moreover, early recognition of cirrhosis enables regular screening for the development of portal hypertension and hepatocellular carcinoma. Many serum tests and scores have been developed, with some of them only available commercially. There are simple tests based on routinely available biochemical markers such as: AST/ALT ratio [6]; BARD score (**B**MI, **A**ST to ALT **R**atio, **D**iabetes mellitus) [7]; NAFLD Fibrosis Score [8]; APRI score (AST to Platelet Ratio Index); and FIB-4 score (based on age, AST/ALT activities and platelet count) [9]. Other scoring systems require special laboratory analyses such as the determination of serum hyaluronic acid (HA) [10], aminoterminal peptide of pro-collagen III (PIIINP), and the tissue inhibitor of matrix metalloproteinase 1 (TIMP-1) levels required for both the OELF (**O**riginal **E**uropean **L**iver **F**ibrosis) [11] and ELF (**E**nhanced **L**iver **F**ibrosis) panels [12].

The aim of our prospective study was to compare the relevance of serum hyaluronic acid levels and other non-invasive scoring systems (APRI, AST/ALT ratio, FIB 4, BARD, NAFLD fibrosis score, ELF, OELF) [8] in the distinguishing of NASH, as well as the staging of liver fibrosis in a group of consecutive NAFLD patients.

## Patients and Methods

### Patients

A total of 112 patients with NAFLD were included in the prospective study. The patient population was recruited from all consecutive patients referred to the 4th Department of Internal Medicine of the General University Hospital in Prague between 2010 and 2013, and in whom a diagnosis of NAFLD had been confirmed. The clinical and laboratory data were either collected from the time of liver biopsy or from the time of confirmation of NAFLD in those patients not indicated for liver biopsy. As inclusion in the study did not involve any especially invasive examination (only blood sampling coupled with the regular blood check), after careful explanation, no patients declined inclusion. The indication for liver biopsy depends solely on the clinical situation and the patient's decision, and was not the determinant factor for inclusion in the study (see paragraph “Liver biopsy”).

The diagnosis of NAFLD was based on liver histology in 56 patients with an available liver biopsy, and on clinical and laboratory parameters in the remainder of the patients. These clinical and laboratory parameters included: 1) elevated levels of ALT, AST, GGT; the presence of steatosis, fibrosis, or cirrhosis on abdominal ultrasound, and 2) a clinical situation compatible with NAFLD (i.e. the presence of metabolic syndrome or certain components of this syndrome), and exclusion of another etiologies of liver disease [13]. Viral hepatitis, drug induced liver disease, autoimmune liver disease, biliary diseases, and inherited metabolic diseases were excluded by specific laboratory and radiologic examinations, as well as the patient history. Alcohol abuse was excluded by the patient's history, short questionnaire, stable GGT activities, and use of either serum carboxyl deficient transferin and/or urine ethyl-glucuronide levels. The presence of an uncontrolled tumor was also an exclusion criterion. The control group for the comparison of the laboratory parameters, inflammatory cytokines, and total antioxidative capacity consisted of 14 healthy individuals. The control population was recruited from the staff of the University hospital in Prague. These individuals were healthy subjects without a history of liver disease or other chronic diseases, and were age- and gender-matched to the patient population. These individuals had normal liver function tests and the same battery of laboratory tests as those patients with NAFLD had that had been used to exclude any liver disease. The study was carried out in full accordance with the Helsinki Declaration, and was approved by the Ethics Committee of the General University Hospital and First Medical Faculty, Charles University in Prague. All subjects had given prior written informed consent.

### Non-invasive liver fibrosis scoring systems

The scoring systems examined for the presence of liver fibrosis were calculated on the basis of following formulae: APRI was calculated as AST (IU/L/upper AST limit/platelet count (×10^9^/L) × 100 [14]; the FIB-4 score [9] according to the formula: age × AST (IU/L/platelet count (×10^9^/L) × ALT (IU/l); the NAFLD fibrosis score [8] according to the formula: −1.675+0.037 × age (yrs) +0.094 × BMI (kg/m2) +1.13 × impaired glucose tolerance or diabetes (yes = 1, no = 0) +0.099 × the AST/ALT ratio −0.013 × platelet count (×10^9^/L) −0.66 × albumin (g/dl); and the BARD score (BMI, AST/ALT Ratio, Diabetes) as the sum of the following three values: BMI>28 = 1 point, AST/ALT >0.8 = 2 points, diabetes = 1 point [7]. The OELF score was calculated using the algorithm: −6.38 − (ln(age) × 0.14)+(ln(HA) × 0.616)+(ln(PIIINP) × 0.586)+(ln(TIMP-1) × 0.472) [11]; whereas, the ELF score was calculated by using the algorithm: −7.412+ (ln(HA) × 0.681)+(ln(PIIINP) × 0.775)+(ln(TIMP-1) × 0.494) [12]. The scoring systems were applied to: 1) the group of 56 patients with a liver biopsy in order to evaluate their sensitivity and specificity for distinguishing NASH and advanced fibrosis; and 2) the group of 56 patients, without a clinically clear indication to have a liver biopsy, in order to evaluate the incidence of NASH and advanced fibrosis in these patients.

### Liver biopsy

A liver biopsy was available in 56 of the patients. In 43 patients, it was conducted by the percutaneous method with a Menghini needle [15], and in another 13 patients by the transjugular method. The indications for transjugular biopsy were obesity, thrombocytopenia, suspicion of liver cirrhosis, and the need for a hepatic venous pressure gradient measurement [16]. The biopsy samples were routinely stained and then read by a single pathologist (JS) who was blind to the clinical and laboratory data. The stage of liver fibrosis was scored based on the Kleiner *et al.* modification [17] of the Brunt *et al*. proposition [18], [19]: stage 0 - no fibrosis, stage 1 - perisinusoidal or portal fibrosis, stage 2 - perisinusoidal and portal/periportal fibrosis, stage 3 - septal or bridging fibrosis, stage 4 - cirrhosis. The indication for liver biopsy reflected usual clinical practice, in particular clinical suspicion of NASH or significant fibrosis. The reasons for not performing a liver biopsy were either the patients rejection of this invasive procedure, or a clinical and laboratory picture of simple steatosis (normal or slightly elevated ALT, typical ultrasound sings).

### Laboratory methods

The biochemical parameters were measured by routine laboratory techniques. Highly sensitive CRP (hs-CRP) was measured by immunonephelometry (Behring Nephelometer II), and inflammatory cytokines (IL-2, IL-6, and TNF-α) by Luminex technology (using multiplex kit from EMD Millipore, Darmstadt, Germany). Adiponectin, leptin, and insulin were measured by the ELISA technique (Roche Diagnostics, Indianapolis, IN, USA). Serum HA was measured using a method of latex agglutination (Hyaluronic acid LT, Latex Agglutination Method, Wako Chemicals GmbH. Germany). M30 and M65 levels were measured by commercially available ELISA tests (PEVIVA AB, Sweden). Blood for laboratory examination was collected on the same day as the biopsy was performed.

### Statistical methods

For sample size determination, we used an estimation based on the AUROC evaluation for single parameters. To reject the null hypothesis, AUC = 0.50 on the 0.05 significance level with the test power at 80%, the sample size is n = 16 in each group.

The results are presented as the mean values with the standard deviation. Either a two-sample t-test or the Mann-Whitney rank test for non-Gaussian distributed variables were used to estimate the intergroup differences. The correlations between different parameters were evaluated by Pearson resp. Spearman correlation coefficient and linear regression. The Chi-square test, resp. Yates's corrected chi-square test was used for frequency table analyses. All tests were two-sided with p<0.05, considered statistically significant. Receiver operating characteristic curve analysis was used to assess the utility of different non-invasive parameters in the discrimination between those patients with/without significant fibrosis or with/without steatohepatitis. The statistical analyses were performed using BMDP Statistical Software (Release 8.1.) and MedCalc software.

## Results

### Characteristics of Patients

NAFLD was diagnosed in 112 patients −79 males and 33 females. Both gender groups exhibited no differences in both the anthropometric, biochemical parameters, and/or the fibrosis stage (data not shown). The male patients had significantly lower ages compared to the females (46.1±14.5 vs 55.6±14.3 years, p = 0.002).

The clinical and laboratory parameters of all patients with NAFLD as well as the controls are given in Table 1. As expected, compared to NAFLD/NASH patients, significantly lower serum glucose, adiponectin, insulin levels, and cytokeratin-18 fragments M30 and M65 levels were observed in healthy controls. On the other hand, no differences in the serum levels of inflammatory markers were detected between both of the examined groups. A liver biopsy was performed in 56 patients.

**Table 1:** Comparison of clinical and laboratory parameters between NAFLD patients and control subjects.

Parameter	NAFLD (n = 112)	Controls (n = 14)	p-value
Age (years)	48.9±14,9	43.9±9	ns
Gender (M/F)	79/33	8/6	ns
Serum glucose fasting (mmol/l)	5.98±1.8	4.7±0.4	<0.001
IL2 (ng/l)	7.35±22.7	10.33±28.3	ns
IL6 (ng/l)	18.37±34.4	6.21±6.7	ns
TNFα (ng/l)	11.84±12	12.08±8	ns
M30 (U/l)	379±375	122±49	<0.001
M65 (U/l)	884±675	301±79	<0.001
hsCRP (mg/l)	26.95±27	15.5±13.5	ns
Leptin (mg/l)	10.82±7.3	9.97±8.7	ns
Adiponectin (mg/l)	6.72±5.2	10.88±5.5	0.002
Insulin (mIU/l))	16.09±11.4	7.59±4.1	<0.001
HA (µg/l)	49.5±103	20.9±15	ns

IL 2: interleukin 2, IL 6: interleukin 6, TNFα: tumor necrosis factor alpha, M30, M65: fragments of cytokeratin-18, hsCRP: high sensitive C-reactive protein, HA: hyaluronic acid, ns: non-significant. The results are given as mean ±standard deviation.

### Differentiation of NASH from simple steatosis

**Figure 1:**
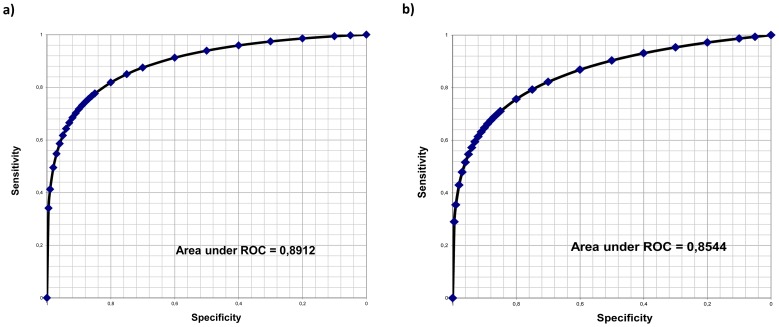
Predictive discrimination of hepatocyte death biomarkers, as determined by ROC plot analysis. The ROC analyses indicate the threshold for M65 (a) and M30 (b) for the best compromise sensitivity/specificity to predict NASH (n = 38 patients) versus steatosis (n = 18 patients) in a group of patients with NAFLD. ROC – receiver operating characteristic curve.

Histological signs of NASH were present in 38 patients, with simple steatosis in the remaining 18 patients. Clinical and laboratory data of these two subgroups are presented in Table 2. In brief, patients with NASH had significantly higher serum levels of GGT, AST, and triglycerides. Patients with NASH had more advanced fibrosis, compared to patients with simple steatosis (mean fibrosis stage 2.64±0.9 vs. 1.3±1.4; p = 0.002); nevertheless, individuals with F3 and F4 fibrosis were also between those patients without NASH (there were 3 patients with cirrhosis). Serum concentrations of cytokeratin-18 fragments M30 and M65 were significantly higher in patients with NASH, compared to patients with simple steatosis. The sensitivity and specificity of cytokeratin-18 fragments for discrimination between patients with and without NASH were calculated and ROC plotted. The most significant parameter for this discrimination was serum concentration of M65 (sensitivity 80%, specificity 82%, with a cut-off value of 750 U/L). The AUROC for M30 was 0.85; for M65 it was 0.89 (Figure 1a, b; Table 3). No other parameter had a similar sensitivity and specificity (Table 3).

**Table 2:** Clinical and laboratory data in patients with NAFLD, and the presence or absence of histological signs of NASH.

Parameter	NASH+ n = 38	NASH- n = 18	p-value
Age (years)	46.4±15	43.6±16	ns
BMI (kg/m^2^)	31.2±3.01	28.9±4.47	ns
GGT (µkat/l)	5.34±4.7	2.3±2.9	0.004
ALT (µkat/l)	2.25±1.6	1.54±1.3	ns
AST (µkat/l)	1.28±0.7	0.79±0.4	0.003
Triglycerides (mmol/l)	3.08±3.4	1.46±0.6	0.011
Histological stage of fibrosis (Kleiner score)	2.64±0.9	1.3±1.4;	0.002
PIIINP (pg/ml)	539±138	618±147	ns
TIMP-1 (ng/ml)	91.6±12.9	69.5±17	0.004
IL2 (ng/l)	2.42±6.9	10.57±34	ns
IL6 (ng/l)	13.86±24.7	18.7±38.2	ns
TNFα (ng/l)	10.27±7.2	10.51±6.4	ns
M30 (U/l)	516±394	181±85	0.001
M65 (U/l)	1333±804	836±437	0.014
hsCRP (mg/l)	22.48±18.7	19.38±22.6	ns
Leptin (mg/l)	9.8±6.6	10.65±6.9	ns
Adiponectin (mg/l)	7.53±9.1	6.75±4.4	ns
Insulin (mIU/l)	23.99±20.1	14.04±8.4	ns
HA (µg/l)	69.9±112	20.3±14.1	0.057
AST/ALT ratio	0.65±0.2	0.72±0.4	ns
APRI	0.86±0.5	0.65±0.5	ns
FIB 4	1.52±0.7	1.86±2.4	ns
NAFLD fibrosis score	−1.73±1.4	−2.31±3	ns
BARD score	1.46±1.1	1.31±1.3	ns

BMI: body mass index, GGT: γ-Glutamyltransferase, PIIINP: aminoterminal peptide of pro-collagen III, TIMP-1: tissue inhibitor of matrix metalloproteinase 1, IL 2: interleukin 2, IL 6: interleukin 6, TNFα: tumor necrosis factor alpha, M30, M65: fragments of cytokeratin-18, hsCRP: high sensitive C-reactive protein, HA: hyaluronic acid, ns: non-significant. The results are given as mean ±standard deviation.

**Table 3:** Sensitivity and specificity of different parameters for differentiation between the patients with and without histological sings of NASH.

Parameter	Cut-off value	Sensitivity	Specificity	95% CI
M30 (U/l)	211	0.79	0.76	0.56–0.93
	234	0.75	0.81	0.50–0.92
M65 (U/l)	790	0.78	0.85	0.48–0.93
	750	0.80	0.82	0.57–0.95
AST (µkat/l)	0.6	0.71	0.55	0.54–0.88
ALT (µkat/l)	1.02	0.71	0.60	0.52–0.85
GGT (µkat/l)	1.66	0.57	0.50	0.28–0.83

M30, M65: fragments of cytokeratin-18, GGT: γ-Glutamyltransferase, CI: confidence interval.

### Differentiation of liver fibrosis

**Figure 2:**
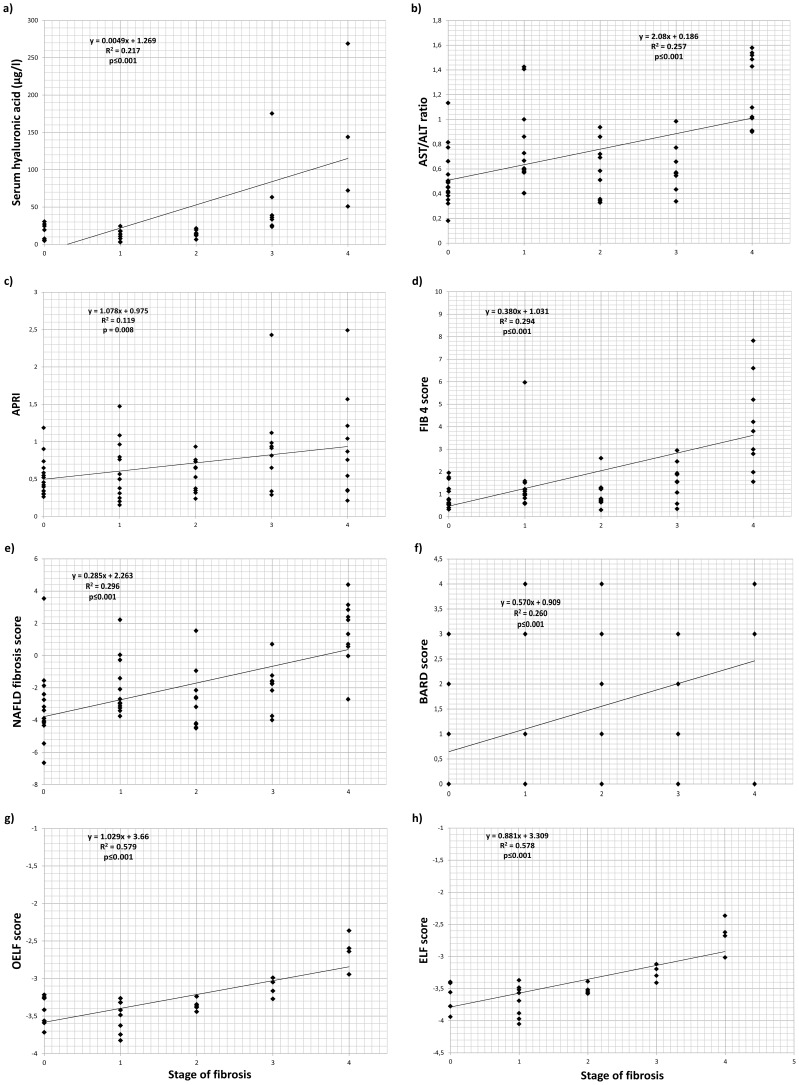
The relationship between serum biomarkers or non-invasive scores and stage of liver fibrosis in 56 patients with biopsy-confirmed NAFLD evaluated by linear regression: Hyaluronic acid (a), AST/ALT ratio (b), APRI score (c), FIB-4 score (d), NAFLD fibrosis score (e), BARD score (f), OELF score (g), and ELF score (h). The non-invasive scores were calculated by formulas given in “Patients and Methods”. The fibrosis stage was evaluated by the Kleiner modification of the Brunt proposition as described in “Patients and Methods”.

The entire group was subdivided into the histological stage of fibrosis (see Table S1). Fibrosis stage F0–F2 was present in 39 patients; F3–F4 in 17 patients. Patients with cirrhosis (F4) were significantly older, compared to other patients (p<0.01). The stage of liver fibrosis unambiguously correlated with the serum concentration of HA (p<0.001; value <75 µg/l excluded the presence of F3/4 fibrosis, Table 4). Similarly, the stage of liver fibrosis correlated with the various fibrosis indexes (AST/ALT ratio, p<0.001; APRI, p<0.01; NAFLD fibrosis score, p<0.001; FIB 4 score, p<0.001; BARD score, p<0.001) (see Figure 2 a–h, Table 4). The stage of liver fibrosis did not correlate with the serum markers of inflammatory reaction (hsCRP, IL2, IL6, p>0.05, Table 4). Congruently, the stage of liver fibrosis did not correlate with the parameters of hepatocyte apoptosis or necrosis (fragments M30 and M65 of cytokeratin-18).

**Table 4:** Correlation between stage of fibrosis and clinical parameters, laboratory parameters, and fibrosis indexes.

Parameter	r	p-value
Age (years)	0.589	<0.001
Thrombocytes (*10^9^/l)	−0.353	<0.01
Albumin (g/l)	−0.320	<0.05
BMI (kg/m^2^)	0.293	<0.05
TIMP-1 (ng/ml)	0.590	<0.01
IL6 (ng/l)	0.331	<0.05
Insulin (mIU/l)	0.366	<0.05
HA (µg/l)	0.486	<0.001
AST/ALT ratio	0.501	<0.001
APRI	0.345	<0.01
FIB4	0.544	<0.001
NAFLD fibrosis score	0.540	<0.001
BARD score	0.510	<0.001
OELF score	0.761	<0.001
ELF score	0.760	<0.001

BMI: body mass index, TIMP-1: tissue inhibitor of matrix metalloproteinase 1, IL 6: interleukin 6, HA: hyaluronic acid.

**Figure 3:**
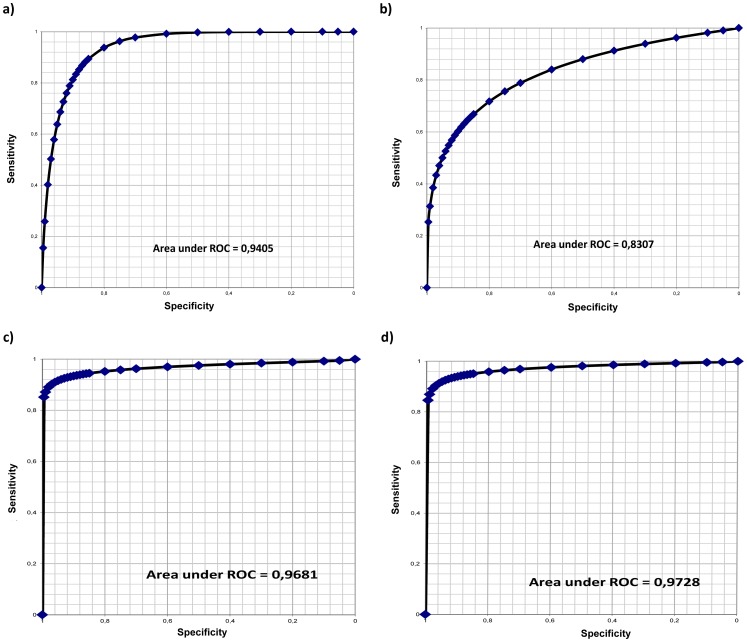
Predictive discrimination of non-invasive fibrosis parameters and scores, as determined by ROC plot analysis. The ROC analyses indicate the threshold for serum hyaluronic acid (a), FIB-4 score (b), OELF (c), and ELF (d) for the best compromise sensitivity/specificity to predict significant fibrosis (F3+F4; n = 17 patients) versus mild or no fibrosis (F0–F2; n = 39 patients) in patients with NAFLD. ROC – receiver operating characteristic curve.

To discriminate between significant liver fibrosis (F3+F4) and mild to moderate or no fibrosis (F0–F2), the sensitivity and specificity were calculated for the different parameters. The most significant parameter for this discrimination was serum HA concentration (sensitivity 80%, specificity 91%, with a cut-off value of 30 µg/l, resp. 90% and 84% with a cut-off value 25 µg/l; AUROC 0.94; Figure 3 a–d, Table 5); OELF score (sensitivity 92%, specificity 93% with a cut-off value of −3.24; AUROC 0.93); and ELF score (sensitivity 90%, specificity 97% with a cut-off value of −3.37; AUROC 0.97). Other parameters did not reach statistical significance (AUROC values were as follows: AST/ALT ratio: 0.73; APRI: 0.70; NAFLD fibrosis score: 0.81; FIB-4∶0.83; and BARD score: 0.77 (Table 5).

**Table 5:** Sensitivity and specificity of different parameters for differentiation between those patients with significant fibrosis (F3,4) and those with none to moderate fibrosis (F0–F2).

Parameter	Cut-off value	Sensitivity	Specificity	95% CI
HA (µg/l)	25	0.90	0.84	0.59–0.99
	30	0.80	0.91	0.44–0.96
FIB-4 score	1.24	0.78	0.72	0.59–0.92
	1.51	0.73	0.78	0.50–0.87
NAFLD fibrosis score	−2.16	0.78	0.70	0.54–0.92
APRI	0.65	0.64	0.66	0.40–0.79
AST/ALT ratio	0.67	0.67	0.67	0.44–0.82
OELF score	−3.24	0.92	0.93	0.58–0.99
ELF score	−3.37	0.9	0.97	0.51–0.99
	−3.39	0.93	0.93	0.51–0.99

HA: hyaluronic acid, CI: confidence interval.

### Comparison of patients with and without liver biopsy

To assess the applicability of the examined non-invasive markers, clinical and laboratory parameters in patients with a liver biopsy and those without a biopsy were compared (Table 6). Based on the non-invasive parameter (M65, cut-off value 750 U/l), patients indicated for a liver biopsy would more frequently have steatohepatitis than those not indicated for liver biopsy (68% vs. 16%, p = <0.001). Regarding liver fibrosis, based on a HA cut-off value of 30 µg/l, significant fibrosis would have a similar proportion of patients not indicated for liver biopsy, compared to those in whom fibrosis was verified histologically (35% vs. 29%, ns).

**Table 6:** Clinical and laboratory data in patients with and without liver biopsy.

Parameter	biopsy +	biopsy −	p-value
Age	44.1±15	52.5±14	**0.007**
BMI	29.6±4.3	30.9±3.3	0.193
GGT (µkat/l)	3±3.5	1.55±1.4	**0.012**
ALT (µkat/l)	1.63±1.3	0.99±0.6	**0.044**
AST (µkat/l)	0.89±0.5	0.65±0.3	**0.030**
Triglycerides (mmol/l)	1.8±1.7	1.8±0.9	0.774
albumin (g/l)	46.1±23.3	46.1±4.9	0.99
Thrombocytes (*10^9^/l)	194±66	242±68	**<0.001**
IL2 (ng/l)	10.46±28.8	2.87±5.6	0.445
IL6 (ng/l)	24±42.2	10.1±14.5	0.310
TNFα (ng/l)	11.4±8.5	12.5±16	0.712
M30 (U/l)	433±373	336±375	**0.021**
M65 (U/l)	1091±742	544±346	**<0.001**
hsCRP (mg/l)	20.9±20.6	32.4±32.6	0.368
Leptin (mg/l)	10.9±7.3	10.8±7.4	0.839
Adiponectin (mg/l)	7.1±6.5	6.3±3.6	0.798
Insulin (mIU/l)	18.3±14.6	14.1±7.1	0.250
HA (µg/l)	57.8±131	37.6±36.4	0.298
AST/ALT	0.72±0.36	0.73±0.25	0.293
APRI	0.67±0.47	0.43±0.35	**<0.001**
FIB 4	1.83±2.1	1.26±0.85	0.575
NAFLD fibrosis score	−2.02±2.8	−2.71±2.2	0.196
BARD score	1.43±1.3	1.5±1.4	0.789

Biopsy +: patients with liver biopsy, biopsy −: patients without liver biopsy, BMI: body mass index, GGT: γ-Glutamyltransferase, IL 2: interleukin 2, IL 6: interleukin 6, TNFα: tumor necrosis factor alpha, M30, M65: fragments of cytokeratin-18, hsCRP: high sensitive C-reactive protein, HA: hyaluronic acid. The results are given as mean ±standard deviation.

## Discussion

There are many reasons to support the use of non-invasive parameters in the diagnoses of NASH/fibrosis instead of an invasive liver biopsy. Apart from the general trend to use non-invasive diagnostic techniques, it is important to note that there is no specific treatment of NASH to be offered to the patients after a confirmation of such a diagnosis by liver biopsy. Even if some treatment could be implemented in the future, the use of a liver biopsy for repeated evaluation of treatment efficacy is inconceivable [20]. Moreover, the diagnostic value of a liver biopsy is frequently overestimated, being burdened with both intra- and inter-observer variability, with an accuracy below 90% [3].

The main challenge in the use of non-invasive methods for NASH and fibrosis detection is their level of applicability and implementation into daily medical practice. Even so, clinicians are faced with various pitfalls. First, some non-invasive methods, and the most specific and sensitive scores, rely on parameters which are not routinely examined and are costly (such as TIMP-1 or PIIINP for OELF and ELF scores) [11], [12]. Secondly, most of the scores and parameters have largely been investigated in cross-sectional studies [8], and their utility in monitoring of the disease's natural history in specific clinical situations (such as screening for fibrosis) is unknown. Additionally, while in the original papers (with definitions of ELF [11] and OELF [12] scores) the formulas gave results in the negative range (or around zero); subsequent papers changed the formula, additionally giving the constant +10 [21], [22]. This fact emphasizes the need for specifying a precise formula in all papers calculating the OELF/ELF score in order to enable a correct reading of the results.

There are two aspects in the evaluation of steatosis/steatohepatitis in patients with NASH. Although the steatosis could be diagnosed by imaging techniques such as ultrasound, its sensitivity is not high enough [23] to discriminate between simple steatosis and NASH; and a liver biopsy is currently required in routine clinical practice. Various biomarkers or scoring systems have been reported to be useful in the detection of NASH. Emerging data suggests that hepatocyte apoptosis, a highly organized and genetically controlled form of cell death, may play an important role in liver injury and disease progression in NAFLD [24]. An increase in hepatocyte cell death by apoptosis is typically present in patients with NASH, but is absent in those with simple steatosis [25]. The most promising non-invasive parameter of NASH seems to be the examination of circulating levels of cytokeratin-18, a biomarker of hepatocyte necrosis and apoptosis [26]. Among the different fragments of cykokeratine-18, M30 (a marker of apoptosis), and M65 (a marker of cell necrosis) have been widely investigated [27]–[29]. Although the AASLD guidelines published in 2012 [13] do not recommend the use of cytokeratin-18 in routine clinical practice, a recent meta-analysis on more than 800 patients in 10 studies [30] concluded that both cytokeratin-18 fragments and total cytokeratin-18 have a clinically meaningful benefit in the non-invasive diagnosing of NASH. In this meta-analysis, the area under the ROC curve for cytokeratin-18 fragments in the identification of NASH was 0.845, with 77% sensitivity and 71% specificity. The authors concluded that CK-18 fragments may be a useful biomarker for screening rather than for identifying NASH. Another recent meta-analysis evaluating the use of serum/plasma cytokeratin-18 fragments described a pooled sensitivity of 66% and specificity of 82% in the diagnoses of NASH [31]. In our study, the relevance of cytokeratin-18 fragments was even higher - the AUROC for discrimination of NASH from simple steatosis in our patients was 0.89 for M65, and 0.85 for M30. Also, the sensitivity and specificity of cytokeratin-18 fragments in the discrimination between simple steatosis and NASH was higher (75% sensitivity and 81% specificity for M30; 80% sensitivity and 82% sensitivity for M65). Based on our results, the assessment of M65, with a cut-off value 750 U/l, is the best simple non-invasive method with which to diagnose NASH. Our results also support the suggestion for the use of M30/M65 in the screening of NASH, as the differences between patients with NASH and controls were statistically significant.

Another important finding in this study was that a single biochemical parameter (HA serum concentration) was able to discriminate between NAFLD patients with or without significant liver fibrosis. HA cut-off values of 20−30 µg/g were similar to the results of other authors [32]; however, our sensitivity and specificity numbers were higher. Using this parameter, acceptable false positive and false negative results were recorded.

Similar results regarding fibrosis detection were recorded with the OELF and ELF scores. However, it remains questionable if specific and costly laboratory examinations used for OELF and ELF (PIINP and TIMP-1) can be transferred into routine daily medical practice. The other scores that were calculated in our study correlated closely to the stage of fibrosis; however, the discrimination values did not reach a satisfactory accuracy, having had low sensitivity and specificity (data not shown). Surprisingly, this was also the case with the NAFLD fibrosis score, which is endorsed by current AASLD guidelines [13] as a screening test to exclude low-risk individuals with fibrosis. The NAFLD fibrosis score [8] has been validated to discriminate between various stages of biopsy-verified NAFLD vs. advanced liver fibrosis to identify patients at the highest risk for progression to end-stage liver disease. In our study, the values of the NAFLD fibrosis score differed between patients with different fibrosis stages, but without having statistical significance. We speculate this was due to our specific population (or to other factors).

There are also novel approaches to build-relevant panels for non-invasive fibrosis assessment; for instance, the use of non-linear machine learning techniques [33]. These systems allow for estimating the variable importance, and therefore can be used to further improve precision performance. Using a “machine learning” technique, combining different parameters of fibrosis and cell death, Sowa JP et al. [33] were able, with adequate accuracy, to retrospectively differentiate even those patients with a fibrosis score of 1 or 2. However, this observation should be evaluated in prospective studies.

Another interesting observation concerns the high percentage of possible advanced fibrosis in patients not indicated for liver biopsy. In our study, liver biopsies confirmed NASH in 38 patients (68% of all patients indicated for liver biopsy). Based on a M65 threshold value of 750 U/l, NASH was probably present in 65% of patients (in 4 cases there would have been a false negative diagnosis, and in 2 cases a false positive diagnosis). To the contrary, in patients not indicated for liver biopsy (based on the M65 threshold value mentioned above) NASH would be diagnosed in only 16% (!!) of patients. Significant fibrosis was described from biopsies in 17 patients (30% of all patients indicated for liver biopsy). When using the simple non-invasive test (HA, threshold of 30 µg/l) significant fibrosis would be considered in 29% of patients (in 3 cases a false negative, in 4 cases a false positive). In patients not indicated for liver biopsy, the same percentage as in the biopsy group (35%) fulfilled the criteria for significant fibrosis. This data could indicate that the basic clinical and laboratory parameters used in routine practice, when considering the indication to liver biopsy, could select for patients having steatohepatitis; however, a remarkable proportion of those patients with liver fibrosis might elude a proper diagnosis. Our observation documents the fact that diagnosis of significant fibrosis, when based on routine liver function tests and standard ultrasonography, is not appropriate.

One of the limitations of our study appears to be the fact that not all patients had a liver biopsy performed. Nevertheless, the group of patients without a liver biopsy, which had the same battery of biochemical and clinical examination as those patients with a biopsy available, is interesting for many reasons. First, worldwide, hepatologists face this problem in their daily practice. This is due to the lack of strict criteria existing for an indication for a liver biopsy, as well as not all patients indicated for liver biopsy being willing to undergo such an invasive procedure. Selection of our patients was not based on randomization, but rather depended on the clinical situation. As the application of non-invasive criteria for NAFLD/NASH/fibrosis diagnosis to these patients showed, the selection directed most of the patients with NASH toward a liver biopsy. Among patients without a biopsy, only 15% patients presented with a M65 value corresponding to NASH. By contrast, when considering the non-invasive diagnosis of fibrosis, an even higher percentage of patients without biopsy (compared to those with biopsy) had values corresponding to significant fibrosis (35%, resp. 29%). This emphasizes the need to follow and accurately examine those patients with suspected NAFLD but not indicated for a liver biopsy. Paradoxically, the fact that biopsy was not performed in all of our patients and that the selection of this group was affected by factors common in routine clinical practice is one of the most interesting findings in our study!

In conclusion, our study supports the use of non-invasive parameters such as HA, OELF and ELF scores, or cytokeratin-18 fragments in diagnosing of NASH/fibrosis in those patients with NAFLD. It should be emphasized that a high percentage of patients not undergoing a liver biopsy (for a variety of reasons) might have significant fibrosis.

## Supporting Information

Table S1 **Clinical and laboratory data in patients with different stages of liver fibrosis.**
(DOC)
