# Supplementary material for: Use of Non-Invasive Parameters of Non-Alcoholic Steatohepatitis and Liver Fibrosis in Daily Practice - An Exploratory Case-Control Study
Source: PLoS One. 2014 Oct 28;9(10):e111551. doi: 10.1371/journal.pone.0111551 (PMC4211730; doi:10.1371/journal.pone.0111551)
Supplement: Table S1 — Clinical and laboratory data in patients with different stages of liver fibrosis. (DOC) [file pone.0111551.s001.doc]

**Table S1.** Clinical and laboratory data in patients with different stages of liver fibrosis.

| Parameter | F0  (n=16) | F1  (n=13) | F2  (n=10) | F3  (n=9) | F4  (n=8) | p-value |
| --- | --- | --- | --- | --- | --- | --- |
| Age (years) | 37.8 ± 13.8 | 42.1 ± 14 | 37.6 ± 10.6 | 46.2 ± 13.3 | 66.6 ± 6 | F4 vs. F0-3** |
| BMI (kg/m^2^) | 27.5 ± 4 | 30.5 ± 3.2 | 28.5 ± 5.2 | 31.3 ± 4.9 | 31.3 ± 3.2 | F4 vs. F0* |
| ALT (µkat/l) | 1.8 ± 0.9 | 1.4 ± 1.3 | 1.8 ± 1.5 | 2.6 ± 1.9 | 0.5 ± 0.2 | F4 vs. F0-3* |
| AST (µkat/l) | 0.8 ± 0.3 | 0.9 ± 0.6 | 0.9 ± 0.5 | 1.3 ± 0.7 | 0.6 ± 0.3 | ns |
| GGT (µkat/l) | 3.6 ± 4.4 | 1.9 ± 2.7 | 2.9 ± 1.7 | 5.3 ± 5.1 | 1.8 ± 1.4 | F4 vs. F3* |
| Triglycerides (mmol/l) | 1.58 ± 0.7 | 2.5 ± 3.4 | 1.6 ± 0.8 | 2 ± 0.9 | 1.4 ± 0.8 | ns |
| Albumin (g/l) | 46 ± 3.6 | 43.6 ± 5.5 | 44.7 ± 5 | 45.8 ± 4.7 | 35.5 ± 12.5 | F4 vs. F0* |
| Thrombocytes (*10^9^/l) | 210 ± 44 | 201 ± 54 | 223 ± 67 | 215 ± 65 | 119 ± 55 | F4 vs. F0-3* |
| PIIINP (pg/ml) | 602 ± 134 | 542 ± 174 | 699 ± 141 | 518 ± 32 | 994 ± 195 | F4 vs. F2-3*  F4 vs.  F0-1** |
| TIMP-1 (ng/ml) | 65 ± 20 | 78 ± 16 | 73 ± 17 | 98 ± 10 | 169 ± 83 | F4 vs. F0-1* |
| IL2 (ng/l) | 0.72 ± 1 | 0.93 ± 0.7 | 17.49 ± 51 | 4.13 ± 9.1 | 16.88 ± 29 | ns |
| IL6 (ng/l) | 5.95 ± 7 | 4.29 ± 2.7 | 23.41 ± 54 | 15.45 ± 32 | 41.9 ± 49 | F4 vs. F0-3* |
| TNFα (ng/l) | 6.44 ± 3.7 | 10.25 ± 4.4 | 10.16 ± 6.3 | 7.23 ± 5 | 15.7 ± 10.9 | ns |
| M 30 (U/l) | 339 ± 292 | 365 ± 202 | 399 v 307 | 643 ± 472 | 392 ± 548 | ns |
| M 65 (U/l) | 834 ± 533 | 992 ± 412 | 871 ± 510 | 1325 ± 751 | 1223 ± 957 | ns |
| hs CRP (mg/l) | 14.56 ± 6.5 | 19.82 ± 21 | 22.05 ± 33 | 26.03 ± 14 | 22.09 ± 11 | ns |
| HA (µg/l) | 21.31 ± 11 | 13.11 ± 6.7 | 20.87 ± 20 | 92.58 ± 129 | 269.5 ± 315 | F4 vs. F0-2*; F0**; F3 vs. F0-2** |
| AST/ALT | 0.52 ± 0.2 | 0.76 ±0.3 | 0.62 ± 0.2 | 0.58 ± 0.2 | 1.25 ± 0.3 | ns |
| APRI | 0.53 ± 0.2 | 0.61 ± 0.4 | 0.55 ± 0.2 | 0.94 ± 0.6 | 0.94 ± 0.7 | ns |
| FIB 4 | 0.88 ± 0.5 | 1.43 ± 1.5 | 1.02 ± 0.6 | 1.58 ± 0.8 | 4.89 ± 3.2 | ns |
| NAFLD fibrosis score | -3.72 ± 3.1 | -2.05 ± 1.7 | -2.73 ± 1.9 | -1.9 ± 1.4 | 1.49 ± 2 | ns |
| BARD score | 0.76 ± 0.9 | 1.23 ± 1.3 | 1.2 ± 1.4 | 1.33 ± 0.9 | 3 ± 1.1 | ns |
| OELF score | -3.44 ± 0.18 | | | -2.88 ± 0.31 | | F0-2 vs. F3-4** |
| ELF score | -3.62 ± 0.21 | | | -2.96 ± 0.37 | | F0-2 vs. F3-4** |

** p≤0.01; *p≤0.05; ns non-significant. The results are given as mean ±standard deviation

BMI: body mass index, GGT: γ-Glutamyltransferase, PIIINP: aminoterminal peptide of pro-collagen III, TIMP-1: tissue inhibitor of matrix metalloproteinase 1, IL 2: interleukin 2, IL 6: interleukin 6, TNFα: tumor necrosis factor alpha, M30, M65: fragments of cytokeratin-18, hsCRP: high sensitive C-reactive protein, HA: hyaluronic acid.
